# How to Prevent and Treat Complications in Facelift Surgery, Part 1: Short-Term Complications

**DOI:** 10.1093/asjof/ojab007

**Published:** 2021-02-10

**Authors:** Nicholas R Sinclair, Demetrius M Coombs, Grzegorz Kwiecien, James E Zins

**Affiliations:** Department of Plastic Surgery, Cleveland Clinic Foundation, Cleveland, OH, USA

## Abstract

This article provides a review of available evidence with regard to short-term complications in facelift surgery. The article reviews both the most common complications and less common, but well-described ones. The goal is to offer objective means to minimize postoperative complications and a guide for treatment when they occur.

Facelift surgery remains one of the most common aesthetic procedures in the United States. In 2019, 68,983 facelifts were performed by members of The Aesthetic Society, making it the sixth most common aesthetic surgery.^1^ A thorough grasp of best practices and an intimate familiarity with facelift complications is perhaps the best means of enhancing patient outcomes.

Although the facelift literature is extensive, much of it remains subjective in nature, and outcomes data are non-validated. This limits its scientific value. The following is an attempt to provide the reader with an objective assessment of facelift complications and the most current techniques for prevention and treatment. This article will focus on short-term complications occurring in the first 30 days after facelift surgery. Part 2 will focus on late facelift complications.

## GENERAL PATIENT CONSIDERATIONS

While a comprehensive review of preoperative patient evaluation is beyond the scope of this paper, identifiable preoperative risk factors will be reviewed.

### Age

A significant proportion of facelift patients are in an older age group. In 2019, 90% of patients undergoing facelift surgery were older than 50 years of age and 35% of patients were older than 65.^1^ With proper preoperative screening, however, patients in the advanced age group can undergo facelift surgery with relatively low risk. A retrospective review of 216 consecutive facelifts compared patients above 65 years to those less than 65 years of age. The authors found that older patients had a higher preoperative American Society of Anesthesiologists (ASA) risk classification but no difference in minor or major postoperative complications. Notably, no patients in either arm had an ASA classification greater than 3.^2^ The authors concluded that the elective nature of the procedure allows for strict preoperative screening to maintain a low complication rate.

### Gender

Numerous studies have documented that male facelift patients are at greater risk for hematoma than their female counterparts.^3-6^ Baker et al initially reported that strict perioperative blood pressure control with clonidine significantly reduced the incidence of hematoma in men.^4^ Since that time, others have corroborated this.^6^ Even with perioperative blood pressure control regimens, hematoma rates remain higher in male patients than in females.

In addition to general medical screening, the most relevant risk factors to identify preoperatively are hypertension, smoking, nonsteroidal anti-inflammatory (NSAID)/aspirin use, and antiplatelet/anticoagulant use. The ramifications of each risk factor will be discussed in detail in the following sections.

## COMMON COMPLICATIONS

### Hematoma

Hematoma is the most common facelift complication requiring operative intervention. A previous meta-analysis of studies performed between 2001 and 2013 found a 1.8% incidence of expanding hematoma following a facelift.^7^ A more recent review of the Tracking Operations and Outcomes for Plastic Surgeons (TOPS) database found a similar hematoma rate of 1.97%.^8^ Risk factors for hematoma and techniques to reduce its incidence have been studied extensively.

#### Perioperative Risk Factors

A 2001 study identified systolic blood pressure (SBP) greater than 150 mm Hg (relative risk 3.6), male gender (relative risk 2.8), aspirin or NSAID intake (relative risk 2.0), and smoking (relative risk 2.0) as significant preoperative risk factors for hematoma formation.^3^ A 2012 study corroborated perioperative aspirin consumption as a significant risk factor for postoperative hematoma even when aspirin is stopped 1 week before surgery. This study also identified body mass index (BMI) greater than 25 as an additional hematoma risk factor.^9^

Elevated SBP is the most common modifiable risk factor for postoperative hematoma. A 2014 retrospective review identified a history of hypertension, a preoperative SBP greater than 160 mm Hg, and an operating room peak SBP greater than 165 mm Hg as predictive risk factors for hematoma.^10^ Strict perioperative blood pressure control, including preoperative clonidine administration, has been shown to reduce hematoma incidence in male patients.^4,6^ Additionally, postoperative blood pressure control with intravenous labetalol and hydralazine significantly decreases the incidence of hematoma in both female and male patients.^11^

While elevated SBP is often a primary process, it may be related to other underlying issues, including pain, agitation, and postoperative nausea and vomiting (PONV). A 2011 study found a reduction in the incidence of postoperative hematoma from 7% to 0% when using multimodal therapy including antihypertensives/anxiolytic (150 µg clonidine), analgesics (1 g acetaminophen), and antiemetics (4 g ondansetron) before extubation rather than in response to symptoms in the recovery room.^12^

#### Intraoperative Risk Factors

Whether the type of facelift performed alters hematoma incidence remains unclear. A recent meta-analysis did not find a statistically significant difference in hematoma incidence when superficial musculoaponeurotic system (SMAS) flap, SMAS plication, and deep-plane techniques were compared.^7^ A more recent metanalysis, however, did find a statistically significant increase in the odds ratio of hematoma requiring operative evacuation for deep plane vs SMAS plication (odds ratio [OR] = 1.68, 95% confidence interval [CI] = 1.04 to 2.71, *P* < 0.05) and SMASectomy vs SMAS plication (OR = 2.64, 95% CI = 1.71 to 4.10, *P* < 0.01).^13^ The addition of submental surgery through an anterior incision has also been found to increase the risk by a factor of 4.3.^3^

Rebound bleeding has been implicated as a major cause of facelift hematoma by multiple authors.^12^,^14-16^ The use of local anesthetic with dilute epinephrine causes vasoconstriction and expedites the facelift dissection. However, at the time of closure, the residual epinephrine effect may mask transected blood vessels. Later in the postoperative period, the epinephrine effect dissipates, unmasking bleeding that may contribute to hematoma development. In a 2-part study of more than 900 patients, Jones and Grover evaluated a wide range of hematoma risk factors.^14^ Part 1 of the study found that compression dressings, surgical drains, fibrin glue, or tumescence did not alter hematoma rate. In part 2, the removal of epinephrine from the tumescence reduced the incidence of hematoma requiring surgical evacuation to zero. There was also a significant reduction in minor hematoma requiring aspiration alone. Their conclusion was that the elimination of epinephrine eliminated rebound bleeding.

An additional technique to reduce rebound bleeding is to perform a “second look closure,” closing the first side of the face only after the dissection is completed on the second side and submental work is done.^12^ This allows additional time for the epinephrine effect to dissipate before hemostasis and closure.

Recent studies have suggested that tranexamic acid (TXA) may be beneficial in reducing facelift bleeding intraoperatively and decreasing surgical time by inhibiting fibrinolysis and clot dissolution.^15,16^ However, evidence that it reduces hematoma rate is currently sparse. The ideal route—intravenous, topical, or subcutaneous infiltration combined with local anesthetic—remains unclear.^15-18^

The placement of drains in facelift surgery is common. In a 2000 survey of all active members of The American Society of Plastic Surgeons (ASPS), 90% reported using closed suction drains in facelift surgery.^19^ In a prospective, randomized, controlled trial, low-pressure suction drains produced a statistically significant reduction in ecchymosis.^20^ They have not, however, been shown to alter hematoma rate.^20-22^

Extrapolating from abdominoplasty techniques,^23,24^ the obliterating dead space with quilting sutures has been used to reduce facelift hematoma. A series of 525 consecutive patients underwent facelift with the placement of a “hemostatic net,” an extensive system of quilting sutures placed externally through the skin and the SMAS.^25^ The initial 120 patients, treated without the hemostatic net, were reviewed retrospectively and had a hematoma incidence of 14%. The following 405 patients were followed prospectively and no hematomas were reported. Of note, the initial retrospective patients received surgical drains, whereas the prospective group did not. Whether this technique will become generally accepted remains unclear at this time.

A variety of fibrin sealants, both autologous and synthetic, have had extensive use in facelift surgery. While they appear to reduce facelift drainage, ecchymosis, and swelling, data supporting a reduction in hematoma are less clear. A small prospective study utilizing aerosolized Hemaseel fibrin glue (Haemacure Corp, Sarasota, FL) found a significant decrease in bruising, swelling, and operative duration with a nonsignificant reduction in hematoma rate.^26^ A large retrospective review found a decrease in hematoma incidence from 3.4% to 0.4% in male patients undergoing a drainless deep-plane facelift with Tisseel Fibrin Glue (Baxter Healthcare Corp, Deerfield, IL).^27^ In a Phase 2 exploratory, randomized, controlled trial, the use of ARTISS (Baxter Healthcare Corp, Deerfield, IL) fibrin sealant produced a statistically significant decrease in postoperative drainage (11.5 vs 26.8 mL) and hematoma rate (0% vs 6%).^28^ The larger Phase 3 follow-up study corroborated the decreased drainage but did not find a statistically significant decline in hematoma formation.^29^ A prospective trial with retrospective controls found a statistically significant decrease in postoperative edema, induration, and ecchymosis with the use of fibrin glue (22% vs 0%) but did not detect a difference in hematoma or seroma formation.^30^ A 2009 meta-analysis of fibrin sealants in facelift surgery identified only 3 randomized, controlled, trials and found a statistically insignificant trend toward decreased 24-hour drainage and reduced ecchymosis.^31^ To summarize, available data strongly suggest that fibrin sealants reduce drainage, ecchymosis, and swelling but do not convincingly support a reduction in hematoma.

#### Hematoma Management

**Figure 1.**
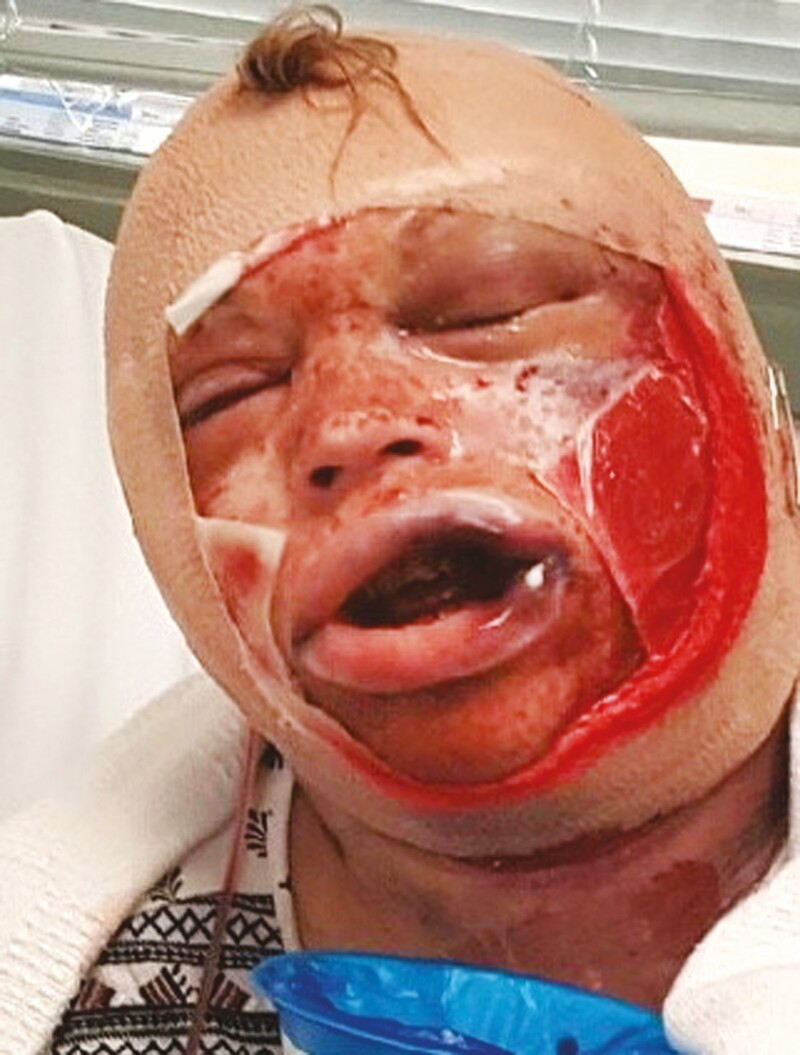
Postoperative appearance of a 61-year-old female who underwent a facelift at an outside hospital. She presented to the Emergency Department approximately 6 hours after discharge with a massive hematoma causing airway compromise. After failed attempts at orotracheal and nasotracheal intubation, she was taken to the operating room for an emergent tracheostomy to manage her airway.

Ninety percent of hematomas occur in the first 24 hours after facelift surgery.^3^ Small, undrained hematomas can cause skin contour irregularities and pigmentation changes. Larger hematomas can threaten skin flap viability and, in rare cases, cause airway obstruction (Figure 1). In general, the treatment for a hematoma following facelift surgery is expeditious surgical evacuation. If there is a delay due to operating room availability or other factors, sutures should be removed to relieve pressure and temporize the situation. Although bedside evacuation of hematoma using a suction catheter has been described,^32^ the safest approach for a sizable collection is a return to the operating room for hematoma evacuation, exploration, and proper identification of areas of bleeding.

#### Our Hematoma Risk Reduction Techniques

In the senior author’s practice (J.E.Z.), the above evidence has been used to formulate a protocol to minimize the risk of hematoma. Patients who take aspirin and other NSAIDs are advised to stop 2 weeks before surgery. Patients who take warfarin, clopidogrel, or other anticoagulants are not considered surgical candidates. Patients with a history of hypertension are optimized before surgery and advised to continue their antihypertensive regimen, including the morning of surgery. The sequence of surgery with regard to rebound bleeding and hematoma prevention is felt to be critical. Approximately, 60 mL of 0.5% lidocaine with 1:200,000 epinephrine and 2 mg of TXA per milliliter of local anesthetic is infiltrated in the subcutaneous tissue of each side of the face. The second look procedure is practiced. That is, the skin dissection, SMAS work, and defatting on the right and left sides of the face are completed, followed by a submental lipectomy and platysmaplasty. Only then are hemostasis and closure completed on the first side, followed by the second side. This allows maximum dissipation of epinephrine effect and minimizes rebound. At the time of closure, the patient’s blood pressure is brought to preoperative baseline in order to further unmask bleeding. Before skin closure, multiple buried quilting sutures are placed from the deep dermis of the skin flap to the SMAS. Closed suction drains are routinely utilized to obliterate dead space and reduce seroma formation. Before extubation, patients receive intravenous acetaminophen for pain and intravenous ondansetron and dexamethasone for PONV. Postoperatively, SBP is strictly maintained below 140 mm Hg with intravenous labetalol and hydralazine.

### Skin Necrosis

Major skin necrosis following facelift surgery is a rare event. However, minor skin slough and delayed healing are not so rare.^33^ Precipitating factors include active smoking, thin skin flaps, excessive tension, undrained hematoma, and wide skin undermining beyond the nasolabial folds.^34^

#### Perioperative Risk Factors

The most common preoperative risk factor for skin necrosis is active tobacco smoking. Rees et al’s retrospective review of 1186 consecutive facelifts documented that active smokers were 12.46 times more likely than nonsmokers to suffer skin slough postoperatively.^35^ A more recent histologic study of skin resected at the time of facelift confirmed these findings. The study demonstrated increased cutaneous occlusive vascular disease in all patients, with specimens from smokers exhibiting significantly more disease than nonsmokers at any given age. Furthermore, the severity of occlusive vascular disease was positively correlated with the incidence of skin slough.^36^

Patients are likely to underreport their smoking status. A 2013 prospective study of 415 patients undergoing various types of plastic surgery found that 4.1% of patients who denied current tobacco use had a positive urine test for the nicotine metabolite, cotinine.^37^ Furthermore, patients who reported that they had quit smoking were significantly more likely to be deceitful than those who had reported that they had never smoked.^37^ This suggests that a preoperative urine cotinine test may be of significant value in the case of former smokers.

The optimal amount of time between smoking cessation and facelift surgery is unknown. Whether the risk of wound healing problems in a former smoker approaches that of a never smoker is also unclear. While 4 weeks is a frequent time interval quoted, data to support a reduction in wound healing problems in facelift surgery could not be found. A search of the available literature identified a retrospective review of patients undergoing free transverse rectus abdominis myocutaneous flap breast reconstruction that provides some evidence-based recommendations. These authors found that smoking-related wound complications (mastectomy flap necrosis and abdominal flap necrosis) decreased when the time interval between smoking cessation and surgery was at least 4 weeks.^38^ Additionally, when such data are discussed, a reduction in wound healing complications rather than a reduction in the overall complications needs to be clarified.

When an active smoker presents for facelift surgery, it is best to advise the patient that smoking cessation before surgery will reduce his or her risk of wound healing complications. Although an optimal time interval is not known, the greater the time between smoking cessation and surgery, the safer the procedure. In these patients, a urine cotinine test is also advisable. A 2000 survey of ASPS members found that approximately 50% of surgeons choose not to perform facelift surgery on active smokers. Plastic surgeons in practice more than 20 years were more likely to operate on smokers compared with plastic surgeons in practice less than 5 years (61% vs 36%).^19^ When performing facelift surgery on a recent smoker, the following is suggested to minimize the risk for wound healing complications: minimize tension, minimize the length of the retroauricular incision, and minimize skin undermining or perform a deep-plane facelift.^34^

#### Intraoperative Risk Factors

While the incidence of skin slough is approximately 3.6% in subcutaneous facelifts, the incidence decreases to less than 1% with a deep-plane technique.^13,33^ In general, a decreased amount of skin flap dissection will decrease the incidence of wound healing issues. Although patients with deep nasolabial folds may require extensive skin undermining beyond the nasolabial fold, this will increase the risk for skin slough.^34^ Excessive tension on the incision leads to hypertrophic scars at best and skin slough at worst. Specifically, to minimize the risk for wound healing issues, there should be no tension on the retroauricular closure.

Combining facelift surgery with skin resurfacing has been suggested to enhance the surgical result. Early work emphasized the need to peel only non-undermined areas when combining chemical peels with facelift surgery.^39,40^ In 1993, a prospective study demonstrated the safety of combining a full-face 35% trichloroacetic acid peel with a deep-plane facelift.^41^

In the past 2 decades, considerable attention has been paid to the combination of facelift surgery and laser skin resurfacing. An initial study in both minipigs and humans suggested that conventional energy level carbon dioxide laser resurfacing at the time of facelift led to delayed healing.^42^ However, more recent clinical series document the safety of deep-plane facelifts or facelifts with limited skin undermining combined with conventional CO_2_, fractionated CO_2_, or erbium laser resurfacing.^43-47^ In reviewing these papers, it appears that resurfacing-related complications are minimized by reducing the extent of skin undermining, using a deep-plane technique, progressively reducing the laser energy delivery from proximal (central face) to distal flap, and lasering only thick, healthy skin flaps.

#### Wound Healing Management

**Figure 2.**
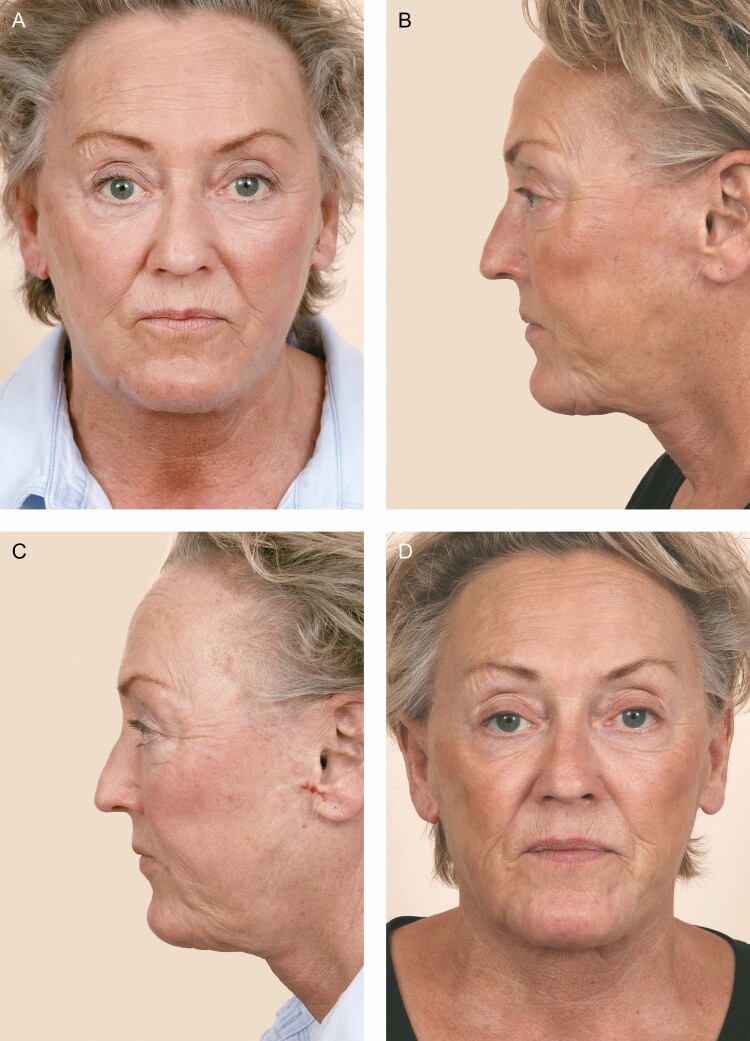
This is a 61-year-old female (A, B) Following hematoma evacuation, the previous patient developed the left side skin slough and delayed healing. She presented to our clinic 3 months following the initial facelift with complaints of residual fullness in her jowls and a widened preauricular scar that had migrated anteriorly. (C, D) One year postoperatively following carbon dioxide laser resurfacing of the left preauricular scar and revision of the submental region with wide undermining and defatting. The fractionated CO_2_ laser was used with the following settings: pattern size 7.6, fractionated coverage 26%, ring size 2, and ring energy 145 mJ. A single pass was made over the preauricular scar on the left and then 2 passes were made on the lobule.

In the absence of an underlying identifiable cause (ie, hematoma or seroma), questionable skin viability should be treated conservatively. The depth of the tissue injury should dictate the choice of dressings. Most of the cases will heal spontaneously with wound care. Secondary scar revision can be performed at a later date (Figure 2).

### Infection

The incidence of surgical site infections following facelift surgery is low. A retrospective review of 6166 consecutive facelifts found a 0.18% incidence of infections requiring hospitalization. The majority of these cases yielded cultures positive for *Staphylococcus* species.^48^ A more recent review of 780 patients undergoing deep-plane facelift found a 0.6% incidence of surgical site infection. Four of the 5 patients with an infection produced cultures that were positive for methicillin-resistant *Staphylococcus aureus* (MRSA), and 2 of those patients required hospitalization.^49^ A 2020 review of the TOPS database identified a 0.89% incidence of infection following facelift surgery.^8^ This study, however, did not identify the depth of infection, causative organism, or treatment. While the reported incidence is low, large retrospective reviews and surveys almost certainly underestimate the true incidence of infection, particularly minor cases of cellulitis.

Although the facelift is considered a clean case, the length of operation alone is an indication for the use of preoperative antimicrobial prophylaxis.^50^ In a 2000 survey of all ASPS members, 72% of respondents used prophylactic antibiotics.^19^ In accordance with the Surgical Care Improvement Project, patients who are not MRSA carriers should receive a single dose of intravenous first-generation cephalosporin 30 to 60 minutes before incision.^50^ To reduce the incidence of postoperative MRSA infections, high-risk groups for MRSA colonization (ie, healthcare professionals) can undergo preoperative nasal culture.^51^ MRSA carriers should then be treated with topical mupirocin ointment for 7 days and chlorohexidine soap body wash for 5 days preoperatively.^52^ If a patient is a known MRSA carrier, vancomycin is the preferred preoperative antibiotic.^50^

There is no evidence to support the use of postoperative antibiotics in facelift surgery. A 2002 survey, however, found that 52% (*n* = 881/1704) of respondents prescribe postoperative antibiotics after facelift surgery.^53^

### Motor Nerve Injury

Although permanent facial nerve injury is rare in the published literature, it is likely underreported. Neuropraxia due to electrocautery or traction injury is more common and is generally temporary.

#### Anatomic Considerations

**Figure 3.**
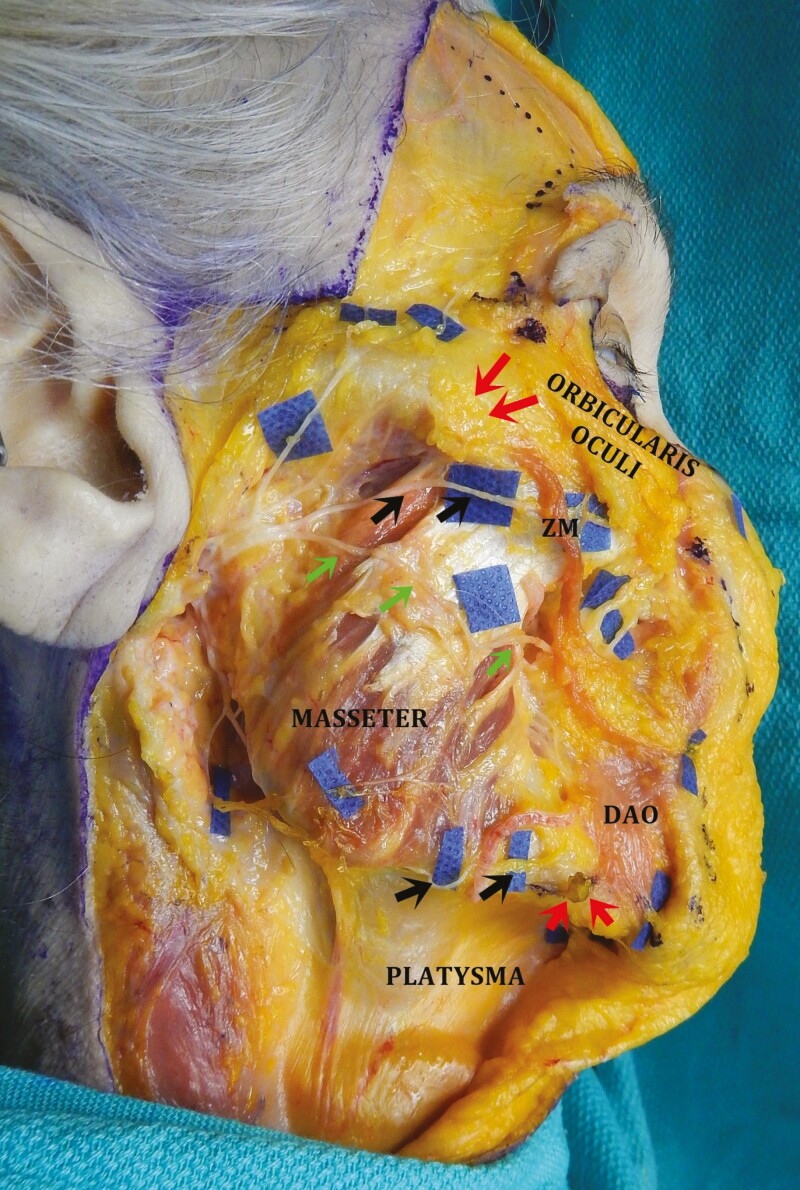
Cadaver dissection of the right hemiface. Skin and subcutaneous tissue have been reflected medially. The SMAS and the parotid gland have been removed and the facial nerve dissected. The zygomatic branch (superior set of black arrows) courses inferior to the zygomatic ligament (superior red arrow) before passing under the *zygomaticus major* (ZM) muscle. This area is referred to as the “sub-SMAS danger zone.” The marginal mandibular branch (inferior set of black arrows) travels anteriorly past the mandibular osseocutaneous ligament (inferior red arrow) to innervate the *depressor anguli oris* (DAO) muscle on its deep surface. The green arrows point to the buccal branch of the facial nerve. The age and gender of the cadaver are unknown.

The buccal branch is the most commonly injured motor nerve in facelift surgery.^54^ However, buccal branch injuries often go unnoticed due to its arborization and cross-innervation with the zygomatic branch. The zygomatic branch is protected by the parotido-masseteric fascia until it passes to the sub-SMAS plane distal to the major zygomatic cutaneous ligament. There are generally 2 branches that pass under the *zygomaticus major* (ZM) muscle: a superior deep branch and an inferior superficial branch. These branches are located approximately 1 cm below the ZM muscle. In this “sub-SMAS danger zone,” ^55^ the nerve is at the greatest risk for injury (Figure 3). In the thin face, the nerve can even be injured during subcutaneous dissection, as the SMAS is quite thin in this area.^56^

**Figure 4.**
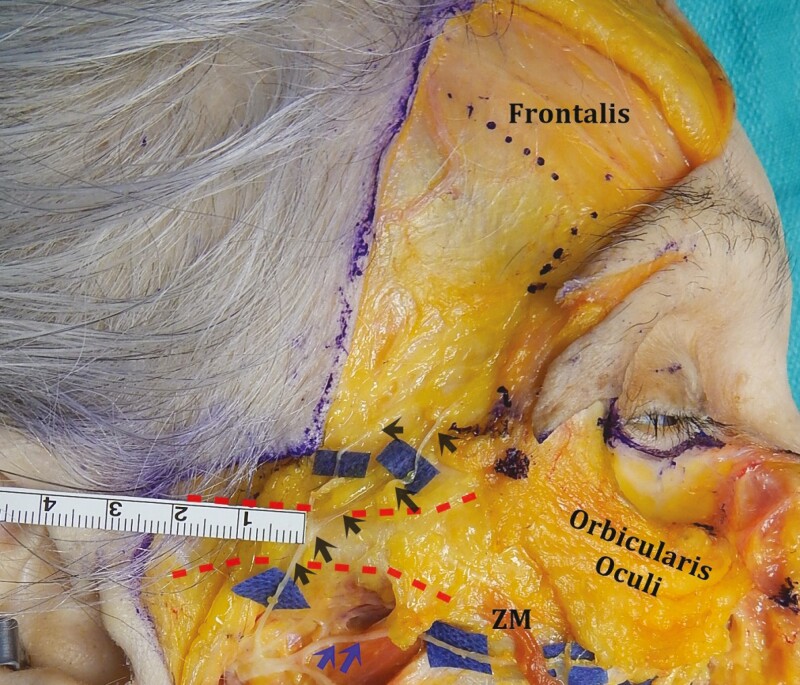
Cadaver dissection of the right hemiface. Skin and subcutaneous tissue have been reflected. After crossing the zygomatic arch (red dashed lines), the frontal branches of the facial nerve (blue background and black arrows) transition from deep (immediately superficial to periosteum) to superficial (immediately deep to the temporoparietal fascia). Note: the zygomatic branches (blue arrows) passing under the *zygomaticus* major (ZM) muscle. The age and gender of the cadaver are unknown.

Injuries to the frontal branch and the marginal mandibular branch are less forgiving due to minimal crossover. The incidence of frontal branch injury is thought to be less than 1%,^57^ and permanent frontal branch dysfunction has been reported to be approximately 0.1%.^19^ The course of the frontal branch can be estimated by Pitanguy’s line, extending from 0.5 cm below the tragus to a point 1 cm above the lateral edge of the eyebrow.^58^ However, variability in brow position, brow ptosis, and cosmetic alterations make this reference point less than ideal. Injury to the frontal branch most likely occurs due to a lack of appreciation of its 3-dimensional (3D) course. The nerve has been shown to consistently cross the zygomatic arch deep to the parotido-masseteric fascia and immediately superficial to the periosteum at a point 4 cm behind the lateral canthus.^59^ Within the first 1.5 to 3 cm superior to the zygomatic arch, the rami become superficial and travel within the lower temporal compartment, immediately deep to the temporoparietal fascia.^60^ It is here that it is most likely to be injured (Figure 4). To minimize the risk of injury, dissection in the region cephalad to the zygomatic arch should be either directly on deep temporal fascia or in the subcutaneous plane.

**Figure 5.**
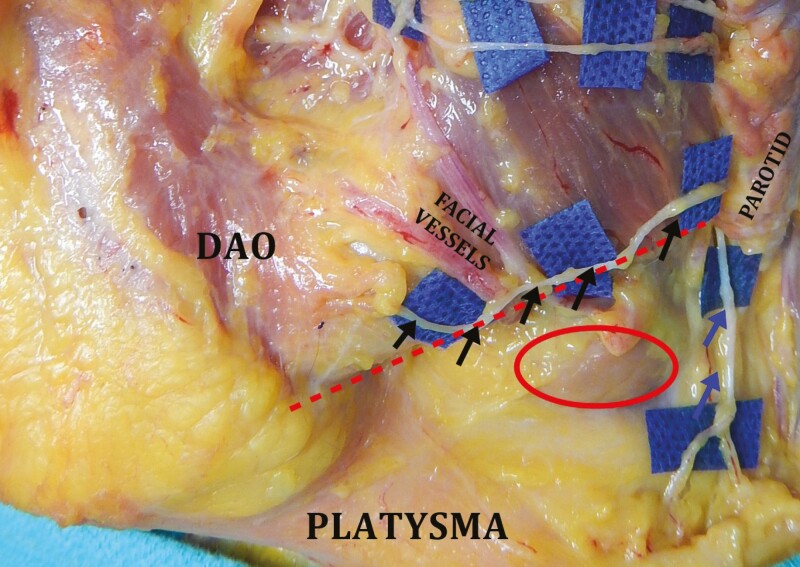
Cadaver dissection of the left hemiface. The inferior mandibular border is marked with a dashed red line. The marginal mandibular branch of the facial nerve (black arrows) can be seen crossing superficial to the facial vessels before innervating the *depressor anguli oris* (DAO) on its deep surface. The cervical branch (blue arrows) courses inferiorly to innervate the platysma. The submandibular gland is delineated with a red ellipse. The age and gender of the cadaver are unknown.

**Figure 6.**
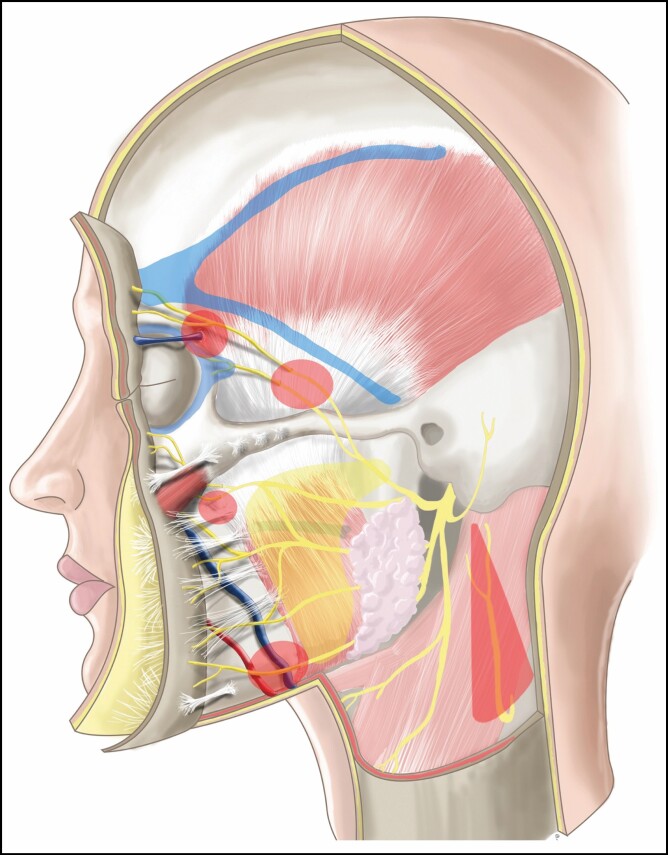
An illustration depicting anatomic landmarks and high-risk areas for facelift surgery. “Danger zones” are shaded in red and “cautious zones” are shaded in yellow. (1) The distal branches of the frontal nerve lie immediately deep to the frontalis muscle, passing superior to the sentinel vein. Dissection in this region should be subcutaneous or subperiosteal. (2) After crossing the zygomatic arch, the frontal rami transition to a more superficial plane, coursing immediately deep to the temporoparietal fascia. Dissection in this region should be subcutaneous or directly on the deep temporal fascia. (3) The zygomatic branches pass deep to the *zygomaticus* major muscle. Sub-SMAS dissection in this region should transition to the subcutaneous plane. (4) The marginal mandibular branch travels in a sub-SMAS plane and passes superficial to the facial vessels. To protect the marginal mandibular nerve, the mandibular osseocutaneous ligament should be released in the subcutaneous plane. (5) After wrapping around the posterior border of the *sternocleidomastoid* muscle, the great auricular nerve courses superiorly in the subcutaneous plane. It travels in Ozturk’s triangle, which is defined by a vertical line drawn through the middle of the lobule perpendicular to Frankfort’s horizontal and a second line drawn 30° posterior from the midpoint of the lobule. Blue shading in the temporal region indicates the superior and inferior temporal septa, which connect anteriorly as the temporal ligamentous adhesion. Blue shading around the orbit indicates the *orbicularis* retaining ligament and lateral orbital thickening. The sentinel vein and facial vein are shown in blue, whereas the facial artery is shown in red.

Given its location in the lower face, the marginal mandibular branch of the facial nerve is at greater risk for injury during facelift surgery than the frontal branch. Again, a thorough understanding of 3D anatomy and emphasis on the prevention of injury is crucial. The marginal mandibular branch exits the parotid deep to the parotido-masseteric fascia. In approximately 80% of patients, the nerve will remain superior to the inferior border of the mandible; however, in 20% of patients, the nerve courses inferior to the lower mandibular border.^61,62^ It then crosses superficial to the facial vessels, but deep to the SMAS, approximately one-quarter of the mandibular length from the gonial angle to the soft tissue pogonion.^63^ When the nerve crosses the facial vessels, it is at the greatest risk for injury and this is considered a facial nerve danger zone. It then continues anteriorly to pass deep to the *depressor anguli oris* (DAO) in a sub-SMAS/platysmal plane, where it is closely associated with the mandibular osseocutaneous ligament (MOCL) (Figures 3, 5, and 6).^63^ To minimize the risk of injury, any MOCL release should be performed in the subcutaneous plane.

**Figure 7.**
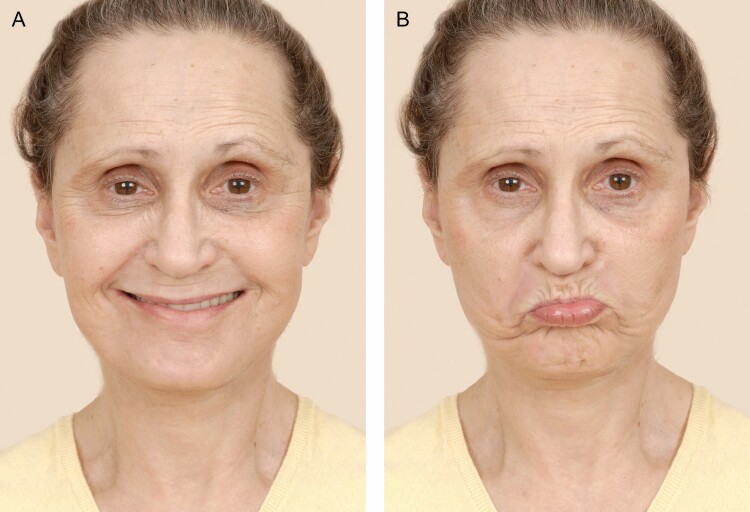
(A) This is a 65-year-old female who underwent an extended SMAS facelift and platysmaplasty through a submental incision. Postoperatively, she was noted to have the right side *depressor anguli oris* (DAO) weakness with a smile. (B) Continued ability to evert her lower lip through intact mentalis function signifies a cervical branch injury, rather a marginal mandibular branch injury. This is known as a “marginal mandibular pseudoparalysis.”

The cervical branch of the facial nerve exits the parotid gland and almost immediately pierces the deep cervical fascia. It passes approximately 1 to 1.5 cm behind the angle of the mandible and then travels anteriorly caudal to the mandible in a subplatysmal plane. When dissecting in a sub-SMAS/platysmal plane, the cervical branch is at greatest risk anterior to the angle of the mandible.^64^ While the cervical branch primarily innervates the platysma muscle, it may also innervate the DAO in a small portion of patients.^65^ In these patients, cervical branch injury can cause a marginal mandibular “pseudoparalysis.” Continued ability to evert the lower lip, through maintained mentalis function, differentiates a true marginal mandibular branch injury from a cervical branch injury (Figure 7).

#### Intraoperative Risk Factors

The type of facelift may increase the risk for temporary nerve injury. A recent meta-analysis found a statistically significant increase in the risk of temporary facial nerve weakness with high lateral SMAS compared with SMAS plication techniques (OR = 2.71, 95% CI = 1.61 to 4.58, *P* = 0.0002) and composite compared with SMAS plication (OR = 2.22, 95% CI = 1.17 to 4.21, *P* < 0.05).^13^ With regard to specific branches, the SMAS flap technique was found to have a significantly reduced odds ratio of temporary frontal branch injury when compared with SMAS plication techniques (OR = 0.21, 95% CI = 0.08 to 0.54, *P* < 0.01) and a reduced OR of temporary marginal mandibular branch injury when compared with SMAS plication (OR = 0.30, 95% CI = 0.14 to 0.63, *P* < 0.01). No difference was found in overall permanent nerve injury when comparing all techniques.^13^

#### Motor Nerve Injury Management

Transient nerve dysfunction and facial asymmetry immediately following the facelift may occur due to residual effects of local anesthetic, which dissipates in hours. Persistent dysfunction after local anesthetic has worn off can be caused by ill-advised suture placement, inadvertent traction, distal SMAS violation, or thermal injury from the use of cautery. Use of bipolar, rather than unipolar, cautery may minimize thermal injury. These issues should resolve without treatment within days or weeks. Nerve dysfunction that persists beyond the initial postoperative period should resolve within 3 to 4 months.^57^ Treatment can include chemodenervation of the contralateral muscle groups with botulinum toxin to achieve better symmetry. Permanent brow ptosis (frontal branch injury) can be definitively treated with a brow lift, while permanent unilateral oral commissure elevation (marginal mandibular branch injury) can be definitively treated with division of the contralateral DAO.

### Sensory Nerve Injury

The great auricular nerve (GAN) is the most commonly injured nerve during facelift surgery with a reported incidence as high as 7%.^66^ To avoid injury, great care should be taken when elevating the lateral postauricular skin flap. The GAN has been classically described as crossing the mid-belly of the *sternocleidomastoid* muscle 6 to 7 cm below the external auditory canal.^67^ Ozturk et al’s triangle is defined by a vertical line drawn through the middle of the lobule perpendicular to Frankfort’s horizontal and a second line is drawn 30° posterior from the midpoint of the lobule (Figure 5). The GAN will invariably lie within this triangle.^68^ Intraoperatively, one can draw this angle as a reference point with brilliant green ink as the skin flap dissection is started below the lobule (see Video). The dissection is kept superficial in this area and the GAN is identified. Once the nerve is located, plication or suspension sutures are placed anterior or posterior to the nerve. If the GAN is transected during flap elevation, it should be repaired under loupe magnification.

While permanent sensory disturbances can occur with GAN transection, the otolaryngology literature documents that even when the GAN is intentionally sacrificed during parotidectomy, the majority of patients regain normal sensation within 1 year.^69^ Furthermore, residual sensory disturbances do not significantly affect the quality of life.^70^ Dysesthesias or neuroma of the GAN is more debilitating and may require surgical exploration for debulking or excision.

### Deep Vein Thrombosis and Pulmonary Embolus

Venous thromboembolism following a facelift is surprisingly rare. In 2006, Reinisch et al reviewed the incidence of deep vein thrombosis (DVT) through a survey of 273 surgeons who performed 9937 facelifts.^71^ They reported 0.35% incidence of DVT and 0.14% incidence of pulmonary embolism (PE). The use of general anesthesia and lack of intermittent compression devices were identified as risk factors. Stuzin et al reported zero clinically evident DVTs or PEs in 10,000 facelifts performed under intravenous sedation with intermittent compression prophylaxis. However, patients were not proactively imaged for DVT or PE.^72^ A 2010 single-center review of 630 patients undergoing facelift found a DVT incidence of 0.31%, a surprisingly similar incidence reported in the Reinisch survey. Both patients had undergone facelift combined with another procedure and both cases lasted longer than 5 hours.^9^ A 2020 database review of 13,346 facelifts identified 2 patients who developed pulmonary emboli (incidence 0.01%).^8^ This study did not provide information regarding DVT prophylaxis or anesthetic modality.

A retrospective review demonstrated that the use of low-molecular-weight heparin (enoxaparin) unnecessarily increased the risk for hematoma (16.2% vs 1.1%) as both the enoxaparin group and control group had zero cases of postoperative DVT or PE.^73^ In this series, surgery was performed under sedation with the use of compression stockings. In response, Stuzin suggests that short operative duration, intravenous sedation, and use of intermittent compression devices were more important than chemoprophylaxis in reducing the risk of facelift-associated thromboembolic disease.^74^

### Parotid Fistula/Sialocele

Parotid fistula and sialocele development following facelift surgery are rare. A 2012 case series and literature review identified 24 reports of cases.^75^ Sialocele following facelift surgery is invariably due to parotid gland injury rather than parotid duct injury. If parotid gland injury is noted intraoperatively, injured tissue can be cauterized and then sealed under the SMAS closure. The reliability of this technique to minimize the risk of parotid fistula is unclear.

The pathognomonic presentation includes recurrent preauricular fluid collection in the first week after surgery. Patients may report increased swelling or drainage when eating. Diagnosis is often clinical but can be confirmed with aspiration and testing for elevated amylase levels. Initial treatment consists of serial aspiration or closed drainage. For persistent cases, intraglandular injection of 100 units botulinum toxin has replaced transdermal scopolamine due to its improved adverse effect profile.^75^

## CONCLUSIONS

Facelift surgery is safe, but certain complications remain well recognized. With adequate patient evaluation, preoperative optimization, a thorough understanding of anatomy, and intraoperative modifications, the risk of complications can be minimized. Honest communication and management of expectations are crucial to maintain patient satisfaction, even in the face of the inevitable complication.

While most of the complications in facelift surgery are not life-threatening and are treated conservatively, postoperative hematoma does require an urgent return to the operating room. No single technique or surgical approach has demonstrated superiority in all patients. Rather, aesthetically pleasing outcomes and safety in facelift surgery require knowledge of the literature, preoperative planning, and meticulous attention to detail.
